# Herpes Zoster Infections in SLE in a University Hospital in Saudi Arabia: Risk Factors and Outcomes

**DOI:** 10.4061/2010/174891

**Published:** 2010-09-13

**Authors:** Afsar Sayeeda, Hussain Al Arfaj, Najma Khalil, A. S. Al Arfaj

**Affiliations:** Division of Rheumatology, Department of Medicine, College of Medicine, King Saud University, P.O. Box 2925, Riyadh 11461, Riyadh, Saudi Arabia

## Abstract

Patients with SLE carry an increased risk of infection that account for 11–23% of all hospitalized patients and 50% of all SLE patients develop major infections during the course of their disease. Globally Herpes Zoster has been reported as the most frequent viral infection in SLE patients. We determined the clinical spectrum, disease sequelae and the risk factors associated with the development of Herpes Zoster in patients with SLE and their outcomes. Retrospective case control study of Herpes Zoster infections was done in SLE patients between 1982 and 2006. Cases were matched 1:2 to controls for age, race, sex and duration of follow up. Clinical features of the cases from the time of lupus diagnosis to the time of Zoster were compared to their respective controls over similar time periods. Thirty two SLE cases were compared to sixty four controls. Cases were more likely to have received cyclophosphamide (*P* = .0223) and intravenous methylprednisolone pulse therapy (*P* = .0026), MMF (*P* < .02), had leucopenia (*P* = .0407) and hemolytic anemia (*P* = .0344). More cases than controls had lupus nephritis, cerebritis, thrombocytopenia but the differences did not reach statistical significance. The mean oral prednisolone dose and proportion of patients receiving immunosuppressives including pulse methylprednisolone therapy, IV Cyclophosphamide and mycophenolate was significantly higher in patients with active SLE compared to patients with SLE in remission at the time of Herpes Zoster (*P* < .05). Disseminated Zoster developed in patients with active SLE (7/9) compared to patients with SLE in remission (0/23). None of the patients had postherpetic neuralgia or bacterial super infection. Immunosuppressive medications were discontinued at the time of diagnosis of Zoster in 19 of 32 patients and all patients received antiviral medications.There were no permanent neurologic deficits or deaths. We conclude that Herpes Zoster infections occur at increased frequency among patients with SLE and carry significant morbidity. Immunosuppressive therapy and severe manifestations of lupus may be risk factors for the development of Herpes Zoster although not necessarily at the time of disease flare or immunosuppressive therapy. Our study suggests that although Herpes Zoster occurs frequently in patients with SLE, it has a relatively benign course.

## 1. Introduction

Herpes Zoster, a painful vesicular eruption with a dermatomal distribution, is caused by reactivation of a latent Varicella Zoster virus infection [1]. It may be complicated by bacterial superinfection and cutaneous and visceral dissemination of lesions which may be life threatening. Although it occurs most commonly in otherwise healthy elderly individuals, immunocompromised patients (i.e., those with malignancies especially lymphoproliferative diseases, organ allograft, and autoimmune diseases such as SLE) appear to be at a particular risk for both Herpes Zoster and its complications [2–8]. Herpes Zoster in these patients has a greater tendency to disseminate and to cause morbidity [9–12]. 

Patients with SLE may be at increased risk for Zoster infection by virtue of both the impairment in cell-mediated immunity which characterizes the disease and by treatment of severe disease with high dose corticosteroids or immunosuppressive agents. Indeed, several studies have reported an increased incidence of Herpes Zoster in patients with SLE [13–18].

There have been no studies in the Saudi population addressing the frequency or natural history of Herpes Zoster infections in patients with SLE or the desirability of withdrawing their immunosuppressive medications and or instituting antiviral therapy. 

In this paper retrospective case controlled study was used to determine its frequency, assess specific risk factors, its association with SLE disease manifestations, and their treatment, and defines prognostic indicators for serious complications of Herpes Zoster.

## 2. Materials and Methods

### 2.1. Patients

SLE patients defined by the ACR 1982 revised criteria seen at the outpatient and inpatient facilities of the university hospital that developed Herpes Zoster were considered for the period extending from 1982 to 2006. Medical records of patients with Herpes Zoster were reviewed to verify the diagnosis and to determine its treatment and outcome as well as the activity of SLE at the time of infection. Patients with Herpes Zoster but with other systemic rheumatic diseases for example, rheumatoid arthritis, dermatomyositis, overlap syndrome, and Wegener's granulomatosis were excluded from this study.

### 2.2. Case Definition and Assessment of Disease

A case was defined as a patient with SLE living or deceased with a history of a characteristic vesicular rash in a dermatomal distribution. Herpes Zoster was considered to be disseminated when the dermatomal pattern was followed by progressive widespread vesicular lesions. Post herpetic neuralgia was defined as pain persisting more than 1 month after resolution of cutaneous lesions.

For the purposes of this study, we included as cases only those patients with a history of Herpes Zoster anytime after the first physician diagnosis of SLE. The diagnosis of Herpes Zoster was made from the history and the documentation of a characteristic rash with associated symptoms in a dermatomal distribution leaving little doubt as to the diagnosis. Viral isolation by culture or serology was not done for confirmation of Herpes Zoster. 

A control was defined as a patient with SLE with no history of Herpes Zoster. Cases and controls were matched 1 : 2 by age, race, gender, and SLE disease duration (from diagnosis to the time of review), clinical manifestations, and treatment of SLE (use of high dose steroid (1 mg/kg/day) & other immunosuppressive agents) in the three months preceding and the three months following the Herpes Zoster episode. Initial disease activity was assessed by the SLE Disease Activity Index score, which is a validated measure of disease activity for patients with SLE [19]. 

Cumulative clinical manifestations of disease and its treatment were compared between patients with and without Herpes Zoster to determine its risk factors. Events were analyzed up to and including the time of the first Herpes Zoster episode in the infected group and to date of the last followup in the uninfected group, to compare disease features during the period when the patients were at risk of first Zoster episode. 

Risk factors were defined by the ever use and dose of prednisolone, use of an immunosuppressive medication, lupus nephritis. The presence of these potential risk factors in cases was determined from the time of SLE diagnosis to the time of Herpes Zoster infection and in controls over similar time periods.

### 2.3. Statistical Analysis

Data were analyzed by SPSS version 12 and the results are presented as percentages and means. Comparison of variables was carried out using chi-square test or Fisher's exact test when appropriate. Statistical significance was defined as *P* < .05.

## 3. Results

A total of 32 cases of Herpes Zoster infection were identified among 624 SLE patients which gives a prevalence of 5.1% of Herpes Zoster infections in SLE. The demographic and clinical features as well as therapy in 32 Herpes Zoster SLE patients were compared with 64 non-Herpes Zoster SLE patients (patients: controls in ratio of 1 : 2) as shown in Table 1.

Table 1 shows that 30 (93.8%) of Herpes Zoster patients were female and 2 (6.3%) were male. Their mean age was 31.4 ± 11.4 years (range 11–60 years) and the mean duration of SLE was 8.6 ± 6.0 years (range 2–22 years). Of them, 30 were Arabs (Saudi and non-Saudi), 1 was Indian, and 1 was Pakistani. The disease was more active in our cases with a mean SLEDAI = 20.7 ± 9.8 (range 2–36) when compared to controls mean SLEDAI = 12.6 ± 5.2 (range 2–29) *P* < .0001.

Regarding the disease manifestations, it was found that nephritis occurred in 18 (56.3%), cerebritis in 2 (6.3%), serositis in 9 (28.1%), leucopenia in 17 (53.1%), thrombocytopenia in 8 (25.0%), and hemolytic anemia in 11 (34.4%) patients. All the 32 patients (100%) received oral prednisolone (PSL), 14 (43.8%) received intravenous cyclophosphamide (Cyclo IV), 16 (50.0%) pulse methylprednisolone (MPSL), 14 (43.8%) azathioprine (AZA), and 6 (18.8%) mycophenolate mofetil (MMF). The mean daily dose of oral PSL was 21.9 ± 27.7 mg (range 5–100 mg). Cyclo IV was given in 12 doses as 10 mg/Kg in 18 months. The mean daily dose of AZA was 125.0 ± 25.0 mg (range 100–150 mg) and of MMF was 1.1 ± 1.1 gm (range 0.5–3 gm). 

The female to male ratio and racial background were similar in the two groups. Herpes Zoster SLE cases were significantly more likely to have had leucopenia (*P* = .04) and hemolytic anemia (*P* = .03) compared to non-Herpes Zoster SLE controls. Although the other serious manifestations including nephritis, cerebritis, serositis, and thrombocytopenia were more frequent in cases compared to controls, the differences did not reach statistical significance (*P* > .05). Immunosuppressives, in particular Cyclophosphamide IV (*P* = .0223) and pulse methylprednisolone (MPSL) (*P* = .0026), were more frequently prescribed to Herpes Zoster SLE patients compared to non-Herpes Zoster SLE patients. 

Herpes Zoster lesions were disseminated in 7 (21.9%) of the 32 patients and localized in 25 (78.1%). Treatment of Herpes Zoster infection comprised of antiviral therapy acyclovir that was administered to 19 (59.5%) patients in oral form and to 13 (40.5%) patients intravenously. SLE was active at the time of Herpes Zoster infection in 9 (28.1%) and in remission in 23 (71.9%) patients. The duration of SLE remission ranged from 2 months to 4 years. SLE did not flare up in response to Herpes Zoster infection. Immunosuppressives were continued in 13 (40.6%) and discontinued in 19 (59.4%) patients. 

Results of SLE treatment regimens at the time of Zoster in relation to disease activity are presented in Table 2. The mean oral PSL dose and proportion of patients receiving immunosuppressives including pulse MPSL, cyclo IV, and MMF was significantly higher in patients with active SLE compared to patients with SLE in remission at the time of Zoster (*P* < .05). 

Disseminated Zoster developed in patients with active SLE (7/9) compared to patients with SLE in remission (0/23). This explains the results in Table 3 that those who developed disseminated Herpes Zoster had received significantly more immunosuppresssives than those who developed localized Herpes Zoster (*P* < .05).

## 4. Discussion

Herpes Zoster reactivation of the Varicella Zoster virus (VZV) occurs in 10%–20% of population over a lifetime [2, 20]. The annual incidence of Herpes Zoster is less than 0.5% in the general population. Herpes Zoster occurs in patients with SLE much more commonly than generally expected [14]. 

Immunological studies on patients with SLE showed that there are basic defects present in the disease itself (i.e., impaired cell-mediated immunity, defective delayed hypersensitivity reaction and a hyperactive humoral immunity). The effects of medication (high dose corticosteroids and immunosuppressive agents or both) used to treat severe disease may further lead to a reduction in the host resistance to infections [21–25]. 

The predisposing risk factors and the nature of complications in SLE patients vary significantly among different ethnic and geographical groups. Japanese patients with SLE seem to be much more susceptible to Herpes Zoster with an incidence of 43% [13, 14], the reported prevalence in different series varying from 13.5% to 46.6% of adult SLE patients. This is much higher than what we found in our population of patients with SLE, the prevalence rate for Herpes Zoster being 6% and by Al-Rayes et al. 5% [26].

Patients who had Herpes Zoster also experienced more episodes of major infections during their disease course [27]. This could be due to disease activity, immunosuppressive therapy and host genetic factors that determine the general susceptibility to both infections and SLE. T cell function plays an important role in maintaining VZV latency in the host. In this study, two thirds of the patients had heightened lupus activity within the 6-month period before Herpes Zoster either because of disease onset or flare up. 

Herpes Zoster lesions were localized in 25% of our patients. The incidence of patients with dissemination in our study was much higher (21.9%) compared to the other studies which ranged from 2%–11% [28, 29]. The reason may be that this is more common among patients receiving high-dose steroids and immunosuppressives [29] or due to a delay in instituting antiviral therapy. The mean dose of oral prednisolone in our SLE cases at the time of Herpes Zoster was 80 mg compared to 12 mg in controls. A reduction in the dose of steroids should be strongly considered in patients receiving high-dose prednisolone at the onset of Zoster episode. 

All our Herpes Zoster patients had uneventful recovery. Two patients had multiple episodes (2 each), and three of them had Herpes Zoster opthalmicus, none of them had clinically apparent CNS or visceral involvement or other complications such as permanent motor deficits, pneumonia, cutaneous scarring, regardless of treatment, or continuation of immunosuppressive medications. None of our patients had post herpetic neuralgia. No patient had bacterial superinfection and there were no deaths. Herpes Zoster in our SLE patients was relatively mild and is similar to that reported by Moutsopoulos et al. in his series [15].

 All our patients received antiviral therapy either in the oral or IV form. Immunosuppressives were discontinued in 59% of our patients but given the duration of action of immunosuppressant agents, altering their regimen will probably be of no benefit [28]. 

In our series and in the series of Khal et al., patients who developed Zoster were more likely to have experienced serious manifestation of SLE, including nephritis, hemolysis, and thrombocytopenia and to have required treatment with cyclophosphamide. In addition, our patients had received intravenous methylprednisolone therapy. The retrospective nature of the study may have led us to miss episodes of Zoster among patients with less serious SLE who might either have had less frequent routine visits or have been less likely to seek medical attention for their Zoster. Moreover, two thirds of our patients had no major SLE organ involvement and were receiving neither high dose steroids nor immunosuprressive therapy in the three months prior to and including the Zoster episode but still sought medical attention. Many of the Zoster reactivations in both these series occurred when SLE was inactive. 

Similar results were shown by Manzi et al. who also noted that the patients who developed Zoster had more severe SLE [16]. They also found immunosuppressive therapy to be a risk factor for the development of Herpes Zoster infections in these patients. Whether immunosuppressive therapy acts as a marker for lupus activity which may be an independent risk factor for Herpes Zoster could not be ascertained from their study. Both bacterial and viral infections have been associated with overall disease activity in hospitalized patients with SLE, independent of immunosuppressive therapy [30].

## 5. Conclusion

We conclude that Herpes Zoster infections occur at increased frequency among patients with SLE and have a relatively benign course. Herpes Zoster infections in patients with lupus are not necessarily associated with excessive morbidity; discontinuing needed immunosuppressive therapy in patients with SLE may be unnecessary in the setting of Zoster infection. Herpes Zoster SLE cases were significantly more likely to have had serious disease manifestations. The mean oral PSL dose and proportion of patients receiving immunosuppressive including pulse PSL, cyclo IV, and mycophenolate was significantly higher in patients with active SLE compared to patients with SLE in remission at the time of Zoster (*P* < .05). Disseminated Zoster developed in patients with active SLE (7/9) compared to patients with SLE in remission (0/23).

Observations by various other authors along with our own suggest that the primary risk for Zoster in patients with SLE is the basic immunologic imbalance intrinsic to lupus, rather than some distinctive immunologic abnormality associated with disease flares or their treatment. Aggressive treatment regimens for lupus, in contrast, are associated with the risk for potentially serious complications of Zoster and patients receiving such regimens should be managed with increased clinical vigilance.

## Figures and Tables

**Table 1:** Comparison of demographic and clinical features in SLE Zoster and SLE Non-Zoster patients.

Characteristics	Cases	Controls	*P* value
	SLE with Zoster (*n* = 32) *N* (%)	SLE without Zoster (*n* = 64) *N* (%)	
Sex			
Male	2 (6.3)	4 (6.3)	
Female	30 (93.8)	60 (93.8)	
Race			.7656
Arabs	30 (93.8)	61 (95.3)	
Non Arabs	2 (6.3)	3 (4.7)	
Nephritis	18 (56.3)	28 (43.8)	.2474
Cerebritis	2 (6.3)	2 (3.1)	.5075
Serositis	9 (28.1)	13 (20.3)	.4089
Leucopenia	17 (53.1)	20 (31.2)	.0407*
Thrombocytopenia	8 (25.0)	11 (17.2)	.3879
Hemolytic anemia	11 (34.4)	9 (14.1)	.0344*
Cyclo IV	14 (43.8)	13 (20.3)	.0223*
MPSL	16 (50.0)	12 (18.8)	.0026*
Azathioprine	14 (43.8)	20 (31.2)	.2336
MMF	6 (18.8)	6 (9.4)	.2318

*significant (*P* < .05), Cylo IV: intravenous cyclophosphamide, MPSL:methyl prednisolone, MMF: mycophenolate mofetil.

**Table 2:** SLE treatment at the time of Zoster infection in relation to SLE activity.

SLE treatment	Active SLE (*n* = 9) *N* (%)	SLE in remission (*n* = 23) *N* (%)	*P* value
MPSL	3 (88.9)	8 (34.7)	.0007*
Cyclo IV	7 (77.8)	7 (30.4)	.0086*
AZA	6 (66.7)	8 (34.8)	.0964*
MMF	6 (66.7)	0 (0.0)	.0002*

*significant (*P* < .05), Cylo IV: intravenous cyclophosphamide, MPSL:methyl prednisolone, AZA: azathioprine, MMF: mycophenolate, mofetil Oral PSL: (Oral prednisolone).

(All patients (100%) received oral prednisolone. The mean dose in active SLE cases was 80 ± 3 mg and in patients with SLE in remission was 12.2 ± 1 mg).

**Table 3:** Disseminated/Localized Zoster in relation to SLE treatment.

SLE Therapy	Disseminated (*n* = 7)	Localized (*n* = 25)	*P* value
	*N* (%)	*N* (%)	
MPSL	6 (85.7)	10 (40.0)	.0094*
Cyclo IV	6 (85.7)	8 (32.0)	.0024*
AZA	5 (71.4)	9 (36.0)	.0808*
MMF	6 (85.7)	0.0 (0.0)	.0000*

*significant (*P* < .05), Cylo IV: intravenous cyclophosphamide, MPSL:methyl prednisolone, AZA: azathioprine, MMF: mycophenolate mofetil.
